# Influence of the hBN Dielectric Layers on the Quantum Transport Properties of MoS_2_ Transistors

**DOI:** 10.3390/ma15031062

**Published:** 2022-01-29

**Authors:** Sara Fiore, Cedric Klinkert, Fabian Ducry, Jonathan Backman, Mathieu Luisier

**Affiliations:** Integrated Systems Laboratory, ETH Zürich, 8092 Zurich, Switzerland; cedrick@iis.ee.ethz.ch (C.K.); fabian.ducry@iis.ee.ethz.ch (F.D.); jbackman@iis.ee.ethz.ch (J.B.); mluisier@iis.ee.ethz.ch (M.L.)

**Keywords:** quantum transport, ab initio, thermal properties, high-k dielectrics, TMD materials

## Abstract

The encapsulation of single-layer 2D materials within hBN has been shown to improve the mobility of these compounds. Nevertheless, the interplay between the semiconductor channel and the surrounding dielectrics is not yet fully understood, especially their electron–phonon interactions. Therefore, here, we present an ab initio study of the coupled electrons and phonon transport properties of MoS${}_{2}$-hBN devices. The characteristics of two transistor configurations are compared to each other: one where hBN is treated as a perfectly insulating, non-vibrating layer and one where it is included in the ab initio domain as MoS${}_{2}$. In both cases, a reduction of the ON-state current by about 50% is observed as compared to the quasi-ballistic limit. Despite the similarity in the current magnitude, explicitly accounting for hBN leads to additional electron–phonon interactions at frequencies corresponding to the breathing mode of the MoS${}_{2}$-hBN system. Moreover, the presence of an hBN layer around the 2D semiconductor affects the Joule-induced temperature distribution within the transistor.

## 1. Introduction

Substantial experimental and theoretical efforts have been invested into the development of novel channel materials that could potentially replace silicon at the end of Moore’s scaling law [1]. The search for suitable dielectric layers has received much less attention, although they might strongly influence the behavior of semiconductor channels [2]. This is especially true for 2D materials and its widely popular transition metal dichalcogenide (TMD) family [3,4]. For example, it has been observed that MoS${}_{2}$ field-effect transistors (FETs) exposed to air exhibit large shifts of their threshold voltage [5,6,7] and a profound degradation of their carrier mobility. These undesired effects, which affect both logic and analog circuit applications, can be alleviated by passivating the active semiconductor material with an oxide layer [8,9,10,11,12]. For practical applications, high-*k* dielectrics such as HfO${}_{2}$ and ZrO${}_{2}$, both with a relative dielectric constant $\epsilon_{R}∼20$, are preferable because they offer a large gate capacitance with low leakage currents [13,14,15]. However, a large hysteresis in the transfer characteristics typically persists because of the high density of dangling bonds and charged impurities at the interface between MoS${}_{2}$ and HfO${}_{2}$ or ZrO${}_{2}$. Moreover, it has been demonstrated that the electron mobility is severely limited by surface optical phonons when a MoS${}_{2}$ monolayer is passivated by a high-*k* dielectric with low-energy polar optical phonons, as encountered in HfO${}_{2}$ or ZrO${}_{2}$ [15]. These polar optical phonon modes can be excited by the electrons in the semiconductor via long-range Coulomb interactions, which become stronger as the semiconductor thickness decreases.

Hexagonal boron–nitride appears as an attractive alternative to HfO${}_{2}$ and ZrO${}_{2}$ to passivate 2D materials [16]. On one hand, it does not induce dangling bonds at the semiconductor–oxide interface. On the other hand, due to the light masses of the nitrogen and boron atoms, its polar optical phonons have a high energy of roughly 200 meV, thus weakening surface optical phonon scattering. In Ref. [15], Ma and Jena theoretically predicted the room-temperature electron mobility of a MoS${}_{2}$ monolayer to be one order of magnitude larger when this semiconductor is embedded within hBN (∼$10^{3}\;$cm${}^{2}$/Vs) rather than SiO${}_{2}$ and HfO${}_{2}$ (∼$10^{2}\;$cm${}^{2}$/Vs).

In addition to these features, hBN possesses a high electronic band gap (∼5eV), a strong mechanical robustness, an excellent thermal stability, chemical inertness, and perfectly clean van der Waals interfaces with transition TMDs. Together with the recent advances in growth techniques [17], these unique properties make hBN an appealing candidate to improve the performance and stability of MoS${}_{2}$ FETs [3,18,19,20,21,22,23,24,25,26,27]. Indeed, experimental studies demonstrated that the trapped charge density is reduced by one order of magnitude when MoS${}_{2}$ is encapsulated within hBN ($1.1\times 10^{11}$ cm${}^{-2}$) instead of SiO${}_{2}$ ($1.9\times 10^{12}$ cm${}^{-2}$) [26]. By comparing the measured temperature-dependent mobility of a MoS${}_{2}$ monolayer encapsulated in a SiO${}_{2}$ [28], HfO${}_{2}$ [29], and hBN dielectric [30], it was found that hBN environments lead to a reduced current hysteresis and the mobility shows a power-law temperature dependence upon cooling, which is the signature of phonon-dominated scattering processes [31].

In terms of heat management, the measured thermal boundary conductance of MoS${}_{2}$/hBN interfaces is equal to 17.0 ± 0.4 MWm${}^{2}$K${}^{-1}$ [27], which compares slightly favorably to the one extracted for MoS${}_{2}$/SiO${}_{2}$, 15.5 ± 1.5 MWm${}^{2}$K${}^{-1}$ [32]. However, the exciting perspectives opened up by hBN are compensated by its relatively low dielectric constant ($\epsilon_{R}∼6$). To obtain the same equivalent oxide thickness (EOT) as with HfO${}_{2}$ or ZrO${}_{2}$, hBN dielectrics must be made 3 times ($\epsilon_{R,HfO_{2}}/\epsilon_{R,hBN}$ = 3) thinner than their high-*k* counterparts. Hence, higher gate leakage currents are expected, above all when the EOT is decreased to 1 nm or below [33,34].

While device simulations have been extensively used to predict the performance of novel channel materials as future, beyond Si-FinFET transistors [35], dielectrics have received much less modeling attention, and their interplay with the underlying semiconductor layer is almost always ignored. Here, we aim to fill this gap and to investigate from first principles the influence of the dielectric environment on the transport properties of MoS${}_{2}$-hBN transistors. Both electron and phonon transport are considered because this combination allows us to more easily identify the vibrational modes that contribute the most to the current reduction. Furthermore, self-heating effects can be taken into account if both the electron and phonon populations are driven out of equilibrium.

The most common approach when simulating a device at the ab initio level consists of including only the active region in the atomic domain. In our case, this corresponds to the MoS${}_{2}$ channel, while the dielectrics embedding it are treated as passive, perfectly insulating, and non-vibrating layers. They only enter the solution of Poisson’s equation, not the Schrödinger one. Such an approach is satisfactory when simulations are restricted to the ballistic limit. To go one step further, thermal vibrations (phonons) should be included. However, phonons cannot be assumed to be confined in the semiconductor channel, as electrons are. These vibrations can propagate through the dielectric so that electrons located in the active region of the transistor can directly couple to them. Therefore, it is necessary to adapt the ab initio simulation domain by incorporating the dielectric layer into it and to explicitly describe its electrical and thermal properties.

With today’s computational resources, the quantum treatment of a semiconductor and of a dielectric layer is not feasible with a realistic thickness (1 nm or more) because the Schrödinger equation scales as the cube of the number of atoms within the device geometry. As a compromise, we restricted ourselves to a simulation domain made of one single layer of hBN stacked on top of a MoS${}_{2}$ monolayer. This configuration will then be compared to the one where only MoS${}_{2}$ is treated at the ab initio level. When the ab initio domain contains both an MoS${}_{2}$ and hBN layer and the electron and phonon populations are driven out of equilibrium, we will show that phonons with an energy larger than the highest MoS${}_{2}$ vibration (∼60meV) are not excited. However, additional phonons induced by the presence of hBN strongly interact with the electron population, especially the breathing mode situated at ℏω=8meV. As a consequence, the scattering rate is increased in this region, which also influences the local lattice temperature.

The paper is organized as follows: Section 2.1 describes how the MoS${}_{2}$-hBN heterostructure is constructed from the unit cells of both monolayers. Section 2.2 presents the calculation of the Hamiltonian and dynamical matrices of the investigated system from first principles. The quantum transport modeling framework and the core equations that are solved are introduced in Section 2.3. Results from device simulation are shown, analyzed, and discussed in Section 3. Finally, conclusions are drawn in Section 4.

## 2. Materials and Methods

### 2.1. Construction of the MoS${}_{2}$-hBN Heterostructure

The modulus of the lattice vectors (*a* and *b*) laying in the plane of the hexagonal cells of hBN and MoS${}_{2}$ measure 2.51 Å and 3.18 Å, respectively. Stacking MoS${}_{2}$ and hBN on top of each other translates into a common supercell that contains a high number of atoms. This number can be limited by compressing the hBN cell size by 0.1%, keeping MoS${}_{2}$ unstrained, and introducing a rotation of 14 degrees to both layers. The resulting supercell is composed of 81 atoms (13 Mo, 26 S, 21 N, and 21 B), with in-plane lattice vectors a=(11.47,0.0,0.0) Å, b=(5.74,9.94,0.0) Å, and an interlayer distance of approximately 3.4 Å, after relaxation of the atomic positions. It should be noted that the strain value is kept very small.

**Figure 1:**
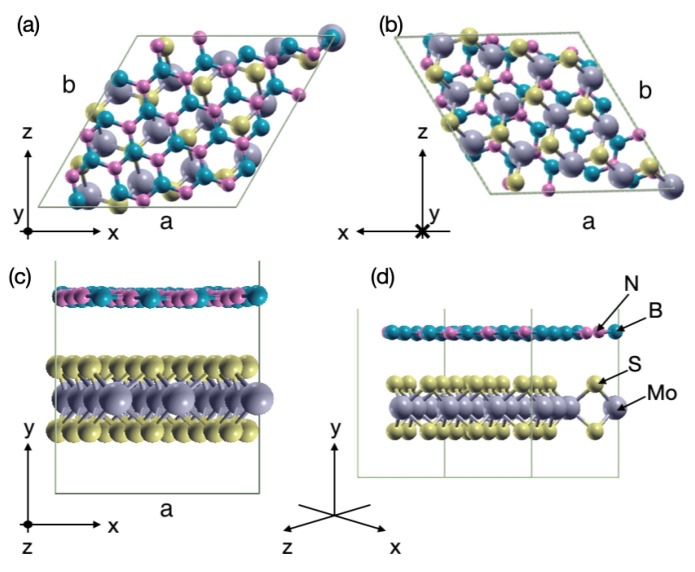
Hexagonal cell built from two MoS${}_{2}$ and hBN monolayers viewed under different angles. The yellow, gray, turquoise, and magenta spheres represent sulfur, molybdenum, boron, and nitrogen atoms, respectively. The *y*-axis refers to the stacking direction, while *x* and *z* are the in-plane axis. (**a**) Top view of the cell. (**b**) Bottom view of the cell. (**c**) Front view of the cell aligned with the *x*-axis. (**d**) Same as (**c**), but rotated of 45 degrees.

The obtained hexagonal cell is plotted in Figure 1. It should be emphasized that such heterostructures do not allow to choose a specific staking order, for example AA’ or AB, contrary to the situation where two TMDs are placed on top of each other or hBN is combined with graphene.

### 2.2. *Ab Initio Calculations*

All the ab initio calculations to obtain the electronic properties of the MoS${}_{2}$-hBN system were performed with the plane-wave (PW) density-functional theory (DFT) package VASP [36,37] using the generalized gradient approximation (GGA) of Perdew, Burke, and Ernzerhof (PBE) [38]. The van der Waals interactions were included through the DFT-D3 method of Grimme [39]. A Γ-centered Monkhorst-Pack *k*-point grid of dimension 7×1×7 and a plane-wave cutoff energy of 550 eV were chosen. Spin-orbit coupling was neglected for simplicity. The convergence criteria for the forces acting on each ion were set to less than $10^{-8}$ eV/Å, whereas the total energy difference between two subsequent iterations had to go below $10^{-10}$ eV. To avoid spurious dipole interactions and coupling between the central unit cell and its periodic replica [40,41], a vacuum layer of 30 Å was placed along the *y*-axis (stacking direction) in Figure 1.

**Figure 2:**
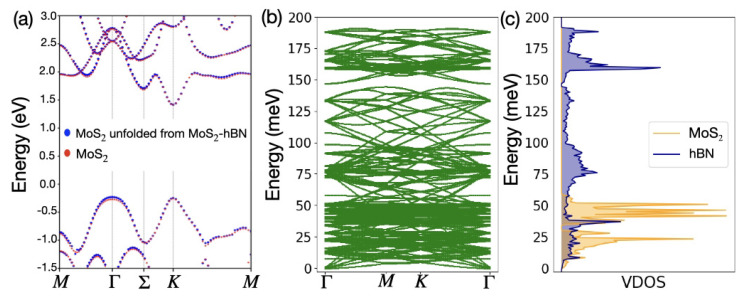
(**a**) Electronic dispersion of an isolated MoS${}_{2}$ (red dots) and the MoS${}_{2}$ layer extracted from the MoS${}_{2}$-hBN heterostructure (blue dots). Both dispersions have been computed with VASP along the same path corresponding to the primitive unit cell of MoS${}_{2}$ via band unfolding, as described in Ref. [42]. (**b**) Phonon dispersion of MoS${}_{2}$-hBN as obtained with CP2K. (**c**) Corresponding vibrational density-of-states (VDOS) projected onto the molybdenum and sulfur atoms (orange area) and onto the boron and nitrogen atoms (blue area).

In Figure 2a, the electronic dispersion of a MoS${}_{2}$ monolayer, computed as described above, is compared to that of the MoS${}_{2}$-hBN heterostructure. To enable a direct comparison, the supercell bandstructure was unfolded along the same path in the Brillouin zone as followed by the pure MoS${}_{2}$ dispersion in the hexagonal unit cell [42]. The interlayer interactions are responsible for a slight lift of the highest valence band (VB) at the Γ point and of the Σ valley in the lowest conduction band (CB). However, the *K* point is only marginally affected. As a consequence, a direct-to-indirect transition is observed. In MoS${}_{2}$-hBN, the band gap occurs between the Γ and *K* points. Its value remains very close (1.64 eV) to the direct one of the pure MoS${}_{2}$ layer (1.67 eV).

To shed light on the nature of the MoS${}_{2}$ and MoS${}_{2}$-hBN sub-bands, we projected the electronic dispersion onto the atomic orbitals. Owing to the large band gap of hBN (∼5eV), the first contribution coming from the B or N atoms in the conduction band appears about 3.5 eV above the CB minimum, which is far beyond the relevant energy range for transport. With respect to MoS${}_{2}$, MoS${}_{2}$-hBN exhibits additional valence sub-bands situated at energies that will not affect the electronic transport properties of the considered device. We found out that these additional bands are associated with the *p*-like orbitals coming from the N atoms.

To perform device simulations at the quantum mechanical level, a localized basis set is desirable because it facilitates the separation of the investigated domain into a central region and two contacts. For this reason, the plane-wave Hamiltonian produced by VASP was transformed into a basis of maximally localized Wannier functions (MLWFs) with the wannier90 tool [43]. Five *d*-like orbitals per Mo atom, three *p*-like orbitals per S and N atoms, and no orbital on the B atoms provide an accurate reproduction of the MoS${}_{2}$-hBN bandstructure around its band gap. The largest energy difference between the MLWF and PW bands around the conduction and valence band edges does not exceed 10 meV.

From the hexagonal unit cells of the MoS${}_{2}$ monolayer and MoS${}_{2}$-hBN heterostructure, the simulation domain corresponding to the device of interest can be constructed by applying the scale-up technique of Ref. [44]. The latter automatically generates the Hamiltonian matrix of the investigated system based on the MLWF data of the original unit cell. For device calculation, an orthorhombic unit cell is often employed as it allows to define a main transport axis that is independent from the directions that are assumed periodic. In case of MoS${}_{2}$-hBN, the orthorhombic cell used for transport is obtained from the hexagonal one by applying the transformation matrix ((1, 0, 0), (1, 2, 0), (0, 0, 1)). The resulting transport cell is composed of 162 atoms and its lattice vector *a*, which is aligned with the *x*-axis (transport direction), has a length equal to 11.47 Å, whereas the lattice vector *b*, aligned with the *z*-axis (periodic direction) and orthogonal to *a*, measures 19.87 Å.

Due to the rotation applied to the atomic planes when the hexagonal MoS${}_{2}$-hBN supercell is constructed, the transport direction is neither aligned with the zigzag nor with the armchair axis. It is shifted by an angle of 14 degrees with respect to the zigzag configuration. However, the obtained transport properties are very close to those obtained in case of a perfect zigzag alignment [45].

Computing the phonon dispersion of the MoS${}_{2}$-hBN supercell is not feasible with VASP due to the heavy computational burden associated with this operation. Therefore, we performed these calculations with CP2K [46], which is a DFT tool that relies on localized Gaussian-type orbitals. When comparing the phonon dispersion of pure MoS${}_{2}$ obtained with VASP and CP2K, a good agreement was found between them, with less than 10% relative difference. This gave us confidence that CP2K can be reliably employed to determine the vibrational properties of the MoS${}_{2}$-hBN system.

To determine the phonon band structure of MoS${}_{2}$, the atomic positions within the unit cell were relaxed with CP2K until the forces acting on each atom were reduced below $10^{-6}$ Hartree/Bohr. A DZVP basis set [47], GTH pseudopotentials [48], and the PBE exchange-correlation functional were selected for that purpose. For the relaxation, a diagonalization algorithm with a *k*-point sampling equal to 2 × 1 × 2 was used. Next, a supercell of dimension 2 × 1 × 2 (324 atoms) was created with PHONOPY [49] from which the required 486 atomic displacements were computed. Due to the large cell size, the calculations of the forces in the supercell were performed at the Γ-point only. The resulting dispersion and corresponding vibrational density-of-states (VDOS) are shown in Figure 2b,c, respectively. As a result of the lighter mass of the B (10.81 amu) and N (14.007 amu) atoms, compared to the one of Mo (95.95 amu) and S (32.06 amu), the phonon spectrum of MoS${}_{2}$-hBN extends over a wider energy range (∼200 meV) than the pure MoS${}_{2}$ (∼60 meV).

### 2.3. Quantum Transport Simulations

**Figure 3:**
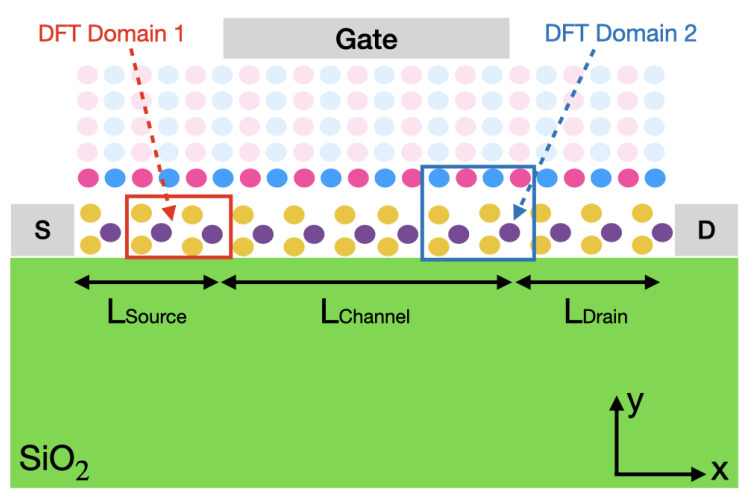
Schematic view of a single-gate monolayer MoS${}_{2}$ field effect transistor. The source, drain, and gate regions measure $L_{Source}=12.5$ nm, $L_{Drain}=12.5$ nm, and $L_{Channel}=15$ nm, respectively. The top and bottom dielectric are hBN and SiO${}_{2}$. The red (cyan) box refers to the DFT domain 1 (2), which contains a MoS${}_{2}$ monolayer (with a hBN single-layer on top of it). The shaded hBN atoms and the SiO${}_{2}$ substrate only enter Poisson’s equation and do not participate in the transport calculation.

To study the influence of hBN on the transport properties of MoS${}_{2}$ FETs, two single-gate device structures were created, one where only the MoS${}_{2}$ monolayer is included in the DFT domain (DFT domain 1) and one where the hBN dielectric environment is also treated at the ab initio level (DFT domain 2), as can be seen in Figure 3. To reduce the computational burden, only one single hBN layer, the one directly on top of MoS${}_{2}$ was included in the DFT calculations, as explained before. The total length of the devices is approximately 40 nm, where the gate and source/drain lengths measure 15 and 12.5 nm, respectively. The source and drain regions are doped with a donor concentration of $2.5\times 10^{13}\;$cm${}^{-2}$, which simultaneously ensures a good electrostatics control and numerical convergence of the simulations up to large gate voltages. Although the chosen doping values are larger than what can be experimentally achieved (∼$10^{13}\;$cm${}^{-2}$) [50,51], we do not believe that the chosen concentrations artificially increase the ON-state of the simulated devices, as demonstrated in Ref. [45]. An abrupt doping profile is applied at the interface between the source or drain and the channel, while the contacts are assumed to be perfectly ohmic (no contact resistance). The TMD–metal contact resistance is currently one of the main limiting factors in 2D-based devices [52,53,54,55,56,57]. It is typically 10 to 100 times larger than in high-performance Si FinFETs [58], but recent experimental studies have shown that it could be reduced in the order of 100’s Ω·μm, which is a value better compatible with practical applications [59].

In terms of geometry, both FET simulation domains incorporate a SiO${}_{2}$ substrate of thickness 20 nm with a relative permittivity $\epsilon_{R}=3.9$, a single layer of MoS${}_{2}$ with $\epsilon_{R}=15.5$, and a hBN oxide with $\epsilon_{R}=6.19$. The values of the dielectric constants were computed as described in Ref. [60]. The thickness of the hBN dielectric was set to approximately 1.6 nm, which corresponds to a five-layer stack. In one configuration (DFT domain 1), all layers are treated as perfectly insulating and non-vibrating materials. They are only included in Poisson’s equation where they are represented by their dielectric constant and their thickness. Alternatively (DFT domain 2), one hBN layer is integrated into the ab initio domain, and the four others only appear in Poisson’s equation, which is solved on a finite element method (FEM) grid.

The OMEN [61] quantum transport simulator was used to compute the device characteristics. It self-consistently solves the Schrödinger and Poisson equations for electrons and a dynamical equation for phonons via the non-equilibrium Green’s function (NEGF) formalism [62]. The following system of equations is computed for electrons.

(1) $$\left(E\cdot I-H_{MLWF}\left(k_{z}\right)-\Sigma^{R}\left(E,k_{z}\right)\right)\cdot G^{R}\left(E,k_{z}\right)=I,$$

(2) $$G^{≷}\left(E,k_{z}\right)=G^{R}\left(E,k_{z}\right)\cdot \Sigma^{≷}\left(E,k_{z}\right)\cdot G^{A}\left(E,k_{z}\right).$$

In the above equations, *I* is the identity matrix, *E* is the electron energy, $k_{z}$ is the electron momentum along the periodic axis *z*, and $H_{MLWF}$ is the up-scaled Hamiltonian matrix expressed in a MLWF basis. The different Green’s functions are the retarted ($G^{R}$), advanced ($G^{A}={\left(G^{R}\right)}^{†})$, lesser ($G^{<}$), and greater ($G^{>}$) components. The symbol ^†^ indicates the Hermitian transposition. All matrices in Equations (1) and (2) have a size $N_{O}\times N_{O}$, where $N_{O}$ is the total number of orbitals in the considered device. The $\Sigma^{≷}$ self-energy contain a boundary $\Sigma^{≷,B}$ and scattering $\Sigma^{≷,S}$ term. The former connects the simulation domain with semi-infinite leads and is computed through contour integral techniques [63]. The latter is limited here to electron–phonon interactions and approximated in two different ways, as discussed later. For simplicity, the retarded component of the scattering self-energy is extracted from the lesser/greater ones as [64].

(3) $$\Sigma^{R,S}∼\frac{\Sigma^{>,S}-\Sigma^{<,S}}{2}.$$

The energy vector *E* over which the Green’s functions and self-energies are evaluated is homogeneously discretized with an interval of 1 meV (2 meV) between adjacent points in ballistic (dissipative) simulations. The periodic direction *z* is modeled via $N_{kz}$ = 11 points, which has been verified to be sufficiently accurate, given the relatively large size of the orthorhombic cell along *z* ($L_{z}=$19.8 Å).

For each bias point, the charge density computed with Equations (1) and (2) is plugged into Poisson’s equation, which in turn updates the electrostatic potential cast into $H_{MLWF}\left(k_{z}\right)$. This self-consistent procedure continues until the relative difference between the electrostatic potentials calculated at two subsequent iterations does not exceed $10^{-3}$. Finally, all simulations were performed at room temperature.

Most 2D materials are characterized by narrow energy sub-bands that may induce non-physical negative differential resistance (NDR) behaviors in the ballistic limit of transport [44,65]. To avoid them, a phenomenological electron–phonon scattering model can be adopted that eliminates the undesired NDR artifacts and provides the quasi-ballistic properties of the device. This model, denominated “pseudo-scattering model”, opens additional conduction channels between the source and the drain contacts of the MoS${}_{2}$ FETs and thus increases the current at high gate-to-source voltages. It relies on the choice of two parameters: a phonon energy ℏω and a deformation potential $D_{e-p}$. The resulting scattering self-energy has the following form

(4) $$\Sigma^{≶,S}\left(E,k_{z}\right)=D_{e-p}^{2}\left(\left(N_{ph}+\frac{1}{2}\pm \frac{1}{2}\right)G^{≶}\left(E+\hbar \omega ,k_{z}\right)+\left(N_{ph}+\frac{1}{2}\mp \frac{1}{2}\right)G^{≶}\left(E-\hbar \omega ,k_{z}\right)\right),$$

where $N_{ph}=1/\left(e^{\hbar \omega /k_{B}T}-1\right)$ is the Bose–Einstein distribution function, $k_{B}$ is the Boltzmann constant, and *T* is the temperature. The phonon energy ℏω and the scattering strength $D_{e-p}$ were set to 40meV and 25 meV in our simulations, respectively. This model accounts for both the back-scattering of electrons toward the source contact (current reduction) and for the opening additional conduction channels (current increase). However, the phonon population remains at equilibrium. Furthermore, as ℏω and $D_{e-p}$ are empirical parameters, no information about the MoS${}_{2}$-hBN interplay is included.

It was found, based on the work carried in Ref. [45], that this combination of values (ℏω = 40 meV and $D_{e-p}$ = 25 meV) allows getting rid of non-physical negative differential resistance behaviors while inducing moderate back-scattering probabilities. Together, the chosen ℏω and $D_{e-p}$ parameters enable probing the quasi-ballistic limit of transport of the simulated devices.

To go one step further, we decided to drive the phonon population out of equilibrium and to self-consistently couple it to the electron density. In such an approach, the phonon population is renormalized each time an electron emits or absorbs a phonon. By comparing the resulting non-equilibrium phonon population to the equilibrium one, it is then straightforward to identify which phonon modes contribute the most to the scattering strength and whether phonons originating from the hBN layer interact with the electrons situated in the MoS${}_{2}$ channel.

Phonon transport is also modeled with the NEGF formalism by solving the following system of equations

(5) $$\left(\omega^{2}\cdot I-\Phi_{CP2K}\left(q_{z}\right)-\Pi^{R}\left(\omega ,q_{z}\right)\right)\cdot D^{R}\left(\omega ,q_{z}\right)=I,$$

(6) $$D^{≷}\left(\omega ,q_{z}\right)=D^{R}\left(\omega ,q_{z}\right)\cdot \Pi^{≷}\left(\omega ,q_{z}\right)\cdot D^{A}\left(\omega ,q_{z}\right).$$

In Equations (5) and (6), *D* is the phonon Green’s function of retarded, advanced, lesser, or greater type, ω is the phonon frequency, $q_{z}$ is the phonon momentum along the periodic direction *z*, and $\Phi_{CP2K}\left(q_{z}\right)$ is the dynamical matrix constructed with PHONOPY from the CP2K DFT inputs. Its size is 3Na×3Na, where Na is the total number of atoms constituting the simulation domain. All phonon–electron interactions are cast into the $\Pi (\omega ,q_{z})$ self energies, which locally modify the phonon density. To complete the modeling picture, the expression for the electron $\Sigma^{≷,S}\left(E,k_{z}\right)$ and phonon $\Pi^{≷,S}\left(E,q_{z}\right)$ scattering self-energies are provided below. The blocks connecting atoms *n* and *m* situated at position $R_{n}$ and $R_{m}$ are given as [66]

(7) $$\begin{array}{cc} \Sigma_{nm}^{≷,S}\left(E,k_{z}\right)= & i\hbar \sum_{\lambda \lambda^{\prime }}\sum_{l_{1}l_{2}}\sum_{q_{z}}\int \frac{d\omega }{2\pi }\nabla_{\lambda }H_{nl_{1}}\times \\ & G_{l_{1}l_{2}}^{≷}\left(E-\hbar \omega ,k_{z}\right)\nabla_{\lambda^{\prime }}H_{l_{2}m}(D_{l_{1}m}^{≷\lambda \lambda^{\prime }}\left(\omega ,q_{z}\right)-\\ & D_{l_{1}l_{2}}^{≷\lambda \lambda^{\prime }}\left(\omega ,q_{z}\right)-D_{nm}^{≷\lambda \lambda^{\prime }}\left(\omega ,q_{z}\right)+D_{nl_{2}}^{≷\lambda \lambda^{\prime }}\left(\omega ,q_{z}\right)), \end{array}$$

(8) $$\begin{array}{cc} \Pi_{nm}^{S,≷\lambda \lambda^{\prime }}\left(\omega ,q_{z}\right)= & 2_{spin}i\sum_{k_{z}}\sum_{l_{3},l_{4}}\int \frac{dE}{2\pi }\times \\ & (\nabla_{\lambda }H_{l_{3}n}G_{nl_{4}}^{≷}\left(E+\hbar \omega ,k_{z}+q_{z}\right)\nabla_{\lambda^{\prime }}H_{l_{4}m}G_{ml_{3}}^{≶}\left(E,k_{z}\right)\\ & -\nabla_{\lambda }H_{l_{3}n}G_{nm}^{≷}\left(\hbar \omega +E,k_{z}\right)\nabla_{\lambda^{\prime }}H_{ml_{4}}G_{l_{4}l_{3}}^{≷}\left(E,k_{z}\right)\\ & -\nabla_{\lambda }H_{nl_{3}}G_{l_{3}l_{4}}^{≷}\left(\hbar \omega +E,k_{z}\right)\nabla_{\lambda^{\prime }}H_{ml_{4}}G_{mn}^{≷}\left(E,k_{z}\right)\\ & +\nabla_{\lambda }H_{nl_{3}}G_{l_{3}m}^{≷}\left(\hbar \omega +E,k_{z}\right)\nabla_{\lambda^{\prime }}H_{ml_{4}}G_{l_{4}n}^{≷}\left(E,k_{z}\right)). \end{array}$$

The index *m* runs over all atoms, λ and $\lambda^{\prime }$ identify the Cartesian coordinates x,y, and *z*, while the $\nabla_{\lambda }H_{nm}$ terms represent the strength of the electron–phonon coupling and is defined as the derivative of the Hamiltonian block $H_{nm}$ connecting the atoms at position $R_{n}$ and $R_{m}$ with respect to the Cartesian direction λ of the bond connecting them. A description of the $\nabla_{\lambda }H$ elements and of their calculation can be found in Ref. [44]. Note that all blocks $\Sigma^{≷,S}$, $G^{≷,S}$, and $\nabla_{\lambda }H_{nm}$ have the same dimensions $N_{orb,n}\times N_{orb,m}$, where $N_{orb,n/m}$ is the number of orbitals on the atom situated at $R_{n/m}$. To keep the treatment of Equation (7) computationally manageable, only the diagonal blocks $\Sigma_{nn}^{≷,S}$ are retained and the momentum coupling was ignored, which corresponds to Γ-like calculations. This simplification is justified by the fact that large unit cells along *z* are employed and the resulting folded phonon dispersion does not vary much along the $q_{z}$ direction. In addition, to ensure energy and electrical current conservation parts of the off-diagonal entries of $\Pi^{≷,S}$ in Equation (8) are accounted for, they are corresponding to the sums over *m* and *n* in Equation (7). Typically, to perform these sums, all neighbors within a cutoff radius of 50 Å from the considered atom are kept.

Equations (1)–(3) and (5)–(8) must be solved self-consistently, since the self-energies $\Sigma^{≷,S}$ and $\Pi^{≷,S}$ depend on the electron $G^{≷}$ and phonon $D^{≷}$ Green’s functions. In this case, both the electron and the phonon populations are driven out of equilibrium. The total energy is conserved, but exchanges between the populations happen. Hence, local variations of the lattice temperature occur that can lead to self-heating effects.

After convergence, the total electrical current flowing between two adjacent orthorhombic cells situated along the transport direction can be numerically evaluated. It is given by

(9) $$I_{d}=\frac{e}{\hbar }\sum_{k_{z}}\sum_{n,m}\int \frac{dE}{2\pi }tr\left(H_{nm}\cdot G_{mn}^{<}\left(E,k_{z}\right)-G_{nm}^{<}\left(E,k_{z}\right)\cdot H_{mn}\right).$$

The energy current is made of two components, an electrical one

(10) $$I_{dE,e}=\frac{e}{\hbar }\sum_{k_{z}}\sum_{n,m}\int \frac{dE}{2\pi }E\cdot tr\left(H_{nm}\cdot G_{mn}^{<}\left(E,k_{z}\right)-G_{nm}^{<}\left(E,k_{z}\right)\cdot H_{mn}\right)$$

and its phonon counterpart

(11) $$I_{dE,ph}=\sum_{q_{z}}\sum_{nm}\int \frac{d\omega }{2\pi }\hbar \omega \cdot tr\left(\Phi_{nm}D_{mn}^{<}\left(\omega ,q_{z}\right)-D_{nm}^{<}\left(\omega ,q_{z}\right)\Phi_{mn}\right).$$

In these equations, the index *n* runs over all atoms situated in one orthorhombic cell, while *m* runs over all atoms located in the next cell along the transport direction. For electrons, the trace operator tr encompasses all orbitals in a given cell, for phonons it goes over all atoms and their three possible directions of vibrations.

## 3. Results and Discussion

**Figure 4:**
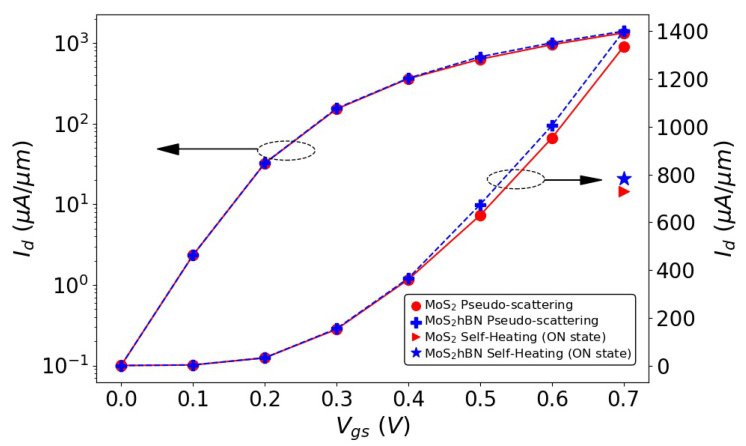
Transfer characteristics $I_{d}-V_{gs}$ at $V_{ds}$ = 0.7 V of the MoS${}_{2}$ FET in Figure 3. The solid line with circles (dashed line with crosses) represents the current for the MoS${}_{2}$ (MoS${}_{2}$-hBN) device when the pseudo-scattering model of Equation (4) is turned on. The triangle (star) indicates the ON-state current of the MoS${}_{2}$ (MoS${}_{2}$-hBN) FET when both the electron and phonon populations are driven out of equilibrium. They are shown on the linear scale corresponding to the right *y*-axis.

**Figure 5:**
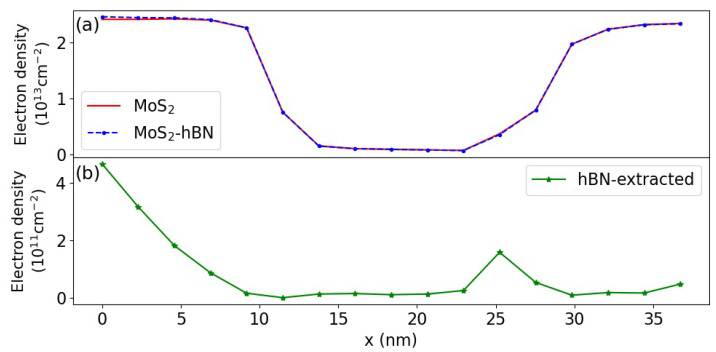
(**a**) Comparison of the electron density in the DFT domain 1 (pure MoS${}_{2}$ device, solid red line) and DFT domain 2 (MoS${}_{2}$-hBN device, dashed blue line with dots) at a gate-to-source voltage $V_{gs}=$0.4 V. (**b**) Electron density in the hBN layer included in the DFT domain 2 at the same gate voltage as in (**a**).

To illustrate the influence of the hBN layer on the MoS${}_{2}$ FET properties, the transfer characteristics of the two devices from Figure 3 are presented in Figure 4, first in the quasi-ballistic limit of transport, i.e., by considering the pseudo-scattering model of Equation (4). The source-to-drain voltage is fixed to $V_{ds}=0.7$ V, while the gate-to-source voltage $V_{gs}$ is ramped between 0 and 0.7 V in steps of 0.1 V. It should be stressed that exactly the same FEM grid was created to solve Poisson’s equation in DFT domain 1 and 2, thus eliminating possible discrepancies coming from this parameter. When examining the $I_{d}-V_{gs}$ curves of both FET configurations of Figure 4, we observe that at low gate voltages, they perfectly agree. As the gate-to-voltage increases, a slight discrepancy can be noticed (4.8% difference in the ON-state current). It can be attributed to the band structures of both material systems, which are not exactly the same, as can be seen in Figure 2. This especially concerns the satellite valleys situated at the Σ point for the CB minimum. Finally, it should be noted that the electron wave functions are centered in the MoS${}_{2}$ semiconductor channel and barely penetrate into the hBN dielectric layer, as illustrated in Figure 5. The electron population in the oxide is about two orders of magnitude lower than in MoS${}_{2}$.

Due to limited computational resources, only the ON-state currents of both device structures (with and without hBN in the DFT domain) could be computed in the presence of out of equilibrium electron and phonon populations. The slow convergence of the scattering self-energies in Equations (7) and (8) explains the difficulty of investigating more bias points under non-equilibrium conditions. To reach current conservation within 2% between the Green’s functions and the scattering self-energy, more than 250 self-consistent Born iterations are needed. The resulting ON-state currents are shown in Figure 4.

**Figure 6:**
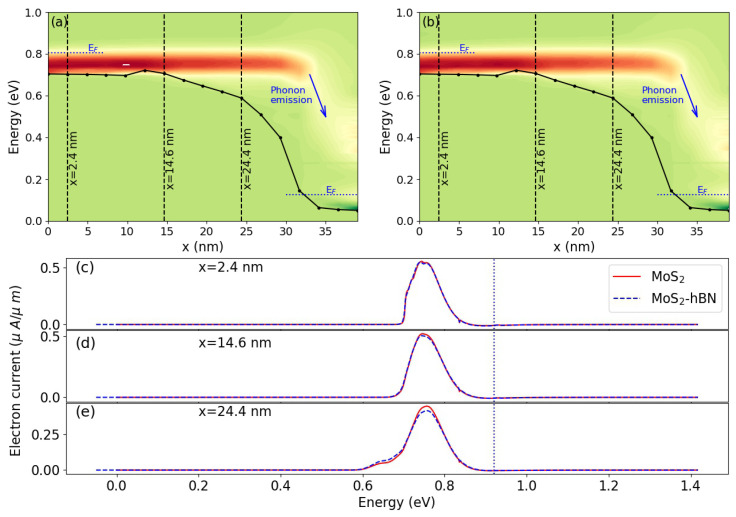
(**a**) Energy- and position-resolved electron current in the DFT domain 1 at $V_{gs}$ = 0.7 V and $V_{ds}$ = 0.7 V in the presence of electron–phonon scattering according to Equations (7) and (8). Red indicates high current concentrations and green indicates no current. The black curve represents the conduction band edge. The dashed blue line close to the source (drain) indicates the Fermi level in the left (right) contact. (**b**) Same as (**a**), but for the MoS${}_{2}$-hBN device (DFT domain 2). (**c**) Energy-resolved electron current at the position x=2.4nm. The solid red line refers to DFT domain 1 in (**a**), the dashed blue line refers to DFT domain 2 in (**b**). The vertical dotted line indicates the position of the Fermi level in the source contact. (**d**) Same as (**c**), but for x=14.6nm. (**e**) Same as (**c**), but for x=24.4nm.

A severe reduction of these quantities as compared to the quasi-ballistic case is observed, about 50%, regardless of the DFT domain. As both FET configurations seem to behave very similarly, we inspected the energy- and position-resolved distribution of the electrical current to identify possible differences between DFT domain 1 and 2. This current is plotted in Figure 6a,b. Here again, no noticeable discrepancy can be detected, as highlighted in Figure 6c–e. In these sub-plots, the electrical current is reported as a function of the energy for the different DFT domains at three positions along the transport direction. Phonon emission and energy dissipation mostly take place close to the drain side of the transistor, as expected, with an almost identical behavior in both cases.

**Figure 7:**
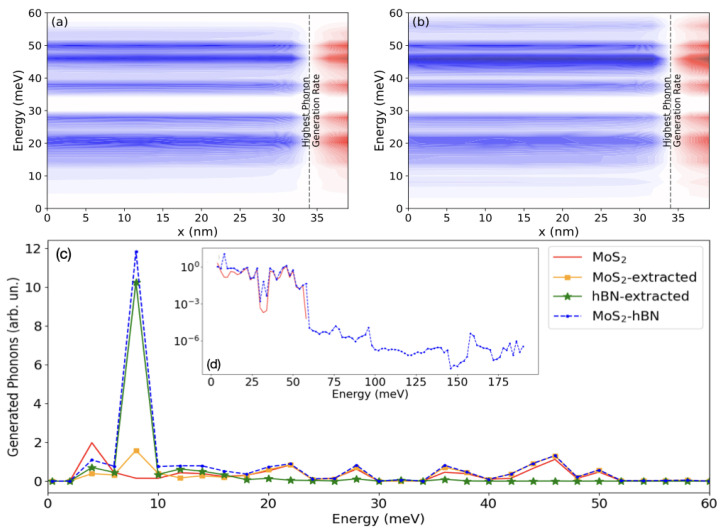
(**a**) Energy- and position-resolved phonon energy current under the same conditions and for the same device as in Figure 6a. Red (blue) indicates positive (negative) currents and white indicates no current. The location of the highest phonon generation rate is marked by a vertical dashed line. (**b**) Same as (**a**), but for the MoS${}_{2}$-hBN device. (**c**) Energy-resolved phonon population generated in the MoS${}_{2}$ FET (solid red line) and in the MoS${}_{2}$-hBN device (dashed blue curve with dots) close to their source contact in the phonon energy range 0<ℏω<60 meV. For the MoS${}_{2}$-hBN structure, the phonons generated in MoS${}_{2}$ (orange curve with squares) are separated from those created in hBN (green curve with stars). The sum of both lines gives the dashed blue line with dots. Inset of (**c**), same as (**c**) but on a logarithmic scale and over the entire MoS${}_{2}$-hBN phonon energy spectrum, i.e., from 0 to 187 meV.

The examination of the energy current carried by phonons in Figure 7a,b is far more revealing. The phonon spectra extend over the same energy range, which means that no high-frequency phonons coming exclusively from the hBN layer (those with ℏω>60meV) are emitted.

To elucidate the origin of this effect, the energy distribution of the phonons generated by emission processes can be visualized. This quantity that indicates the phonon modes that are the most likely to interact with electrons can be calculated by taking the difference between the non-equilibrium and the equilibrium phonon population, where the contributions coming from all $k_{z}$ are summed up. This difference is nothing else but the number of phonons that are created/annihilated through emission/absorption processes at each phonon energy. The non-equilibrium phonon population is the result of the self-consistent electro-thermal simulation, while the equilibrium one assumes a Bose–Einstein distribution function and corresponds to the solution of Equations (5) and (6) when $\Pi^{≷,S}=0$. Figure 7c and its inset report this phonon generation rate. In case of the MoS${}_{2}$-hBN FET (DFT domain 2), the phonons created in the MoS${}_{2}$ channel and in the hBN dielectric have been separated to isolate the influence of each individual layer.

The phonons generated in DFT domain 1 and 2 mostly differ around ℏω=8meV and are very similar in the rest of the spectrum. From the inset of (c) in Figure 7, it can be observed that the phonon generation rate above 60 meV in DFT domain 2 is more than three orders of magnitude smaller than in the 0<ℏω<60meV range, which indicates that electrons do not interact with the pure hBN vibrations. Recalling the behavior of the electron density in Figure 5, this makes sense, as the electronic wave function does not really penetrate into the hBN layer. It is worth investigating what happens at ℏω=8meV, where the largest difference happens. At that specific point, and only at that one, phonons are mostly created inside the hBN layer and with a rate that is about six times larger than the MoS${}_{2}$ contribution. This might be surprising, as the electron wave function does not penetrate into this dielectric, as illustrated in Figure 5.

**Figure 8:**
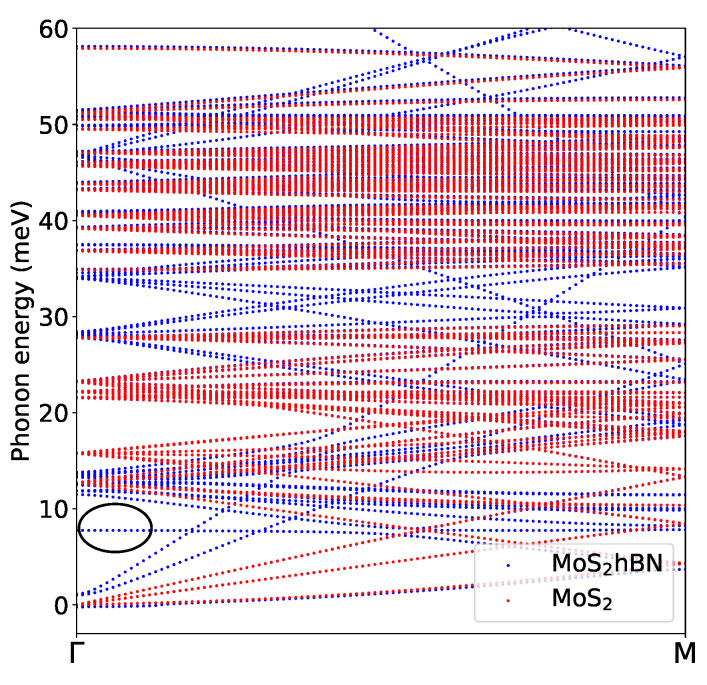
Phonon dispersion of MoS${}_{2}$-hBN (blue dots) and pure MoS${}_{2}$ (red dots) as obtained with CP2K in the energy range 0<ℏω<60meV. The breathing mode at ℏω=8meV is highlighted by a black circle.

**Figure 9:**
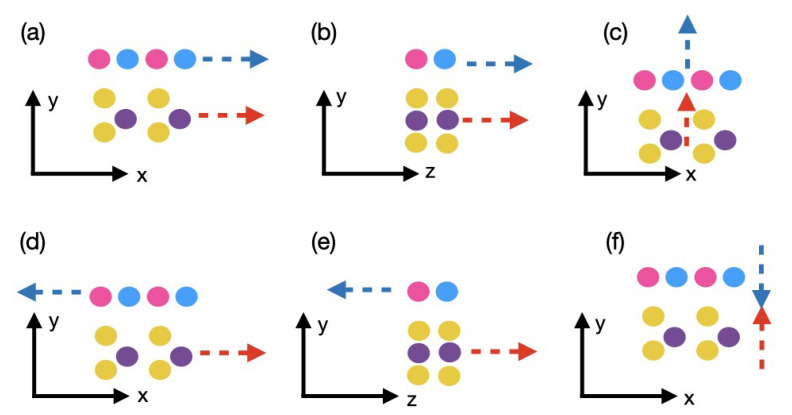
Sketch of the first six phonon modes of MoS${}_{2}$-hBN. The yellow, violet, blue, and magenta spheres represent sulphur, molybdenum, boron, and nitrogen atoms, respectively. The red (blue) dashed arrow represents the direction of displacement of MoS${}_{2}$(hBN). (**a**) LA mode: the two atomic planes move in phase along the *x*-axis. (**b**) TA mode: same as (**b**), but along the *z*-axis. (**c**) ZA mode: same as (**a**), but along the *y*-axis. (**d**) First shearing mode: the two atomic planes move with opposite phase along the *x*-axis. (**e**) Second shearing mode: same as (**d**), but along the *z*-axis. (**f**) Breathing mode: same as (**d**), but along the *y*-axis.

The high phonon generation in hBN at ℏω=8meV can be attributed to the emergence of an isolated mode, known as the breathing mode, which is the sixth one from the bottom in the MoS${}_{2}$-hBN phonon dispersion, as illustrated in Figure 8. The three lowest branches correspond to the LA, TA, and ZA modes, where the two atomic planes move with the same phase along the Cartesian axis. The three next ones involve rigid oscillations of the MoS${}_{2}$ and hBN layers, again along the Cartesian axis, but this time with opposite phase. The fourth and the fifth are known as the first and second shearing modes. The breathing mode is the one corresponding to rigid shifts along the stacking direction, where the two materials move one against each other. With respect to the first five modes, the vibrations of the breathing mode reduce the interlayer distance between the MoS${}_{2}$ and hBN layer of 0.2 Å. Thus, a shorter distance between both 2D layers when they vibrate explains the boost in the phonon generation rate in hBN at ℏω= 8 meV. A sketch of the vibrations of the first six modes is presented in Figure 9. Going back to the phonon generation in Figure 7c and its inset, differences exist at other phonon energies between the pure MoS${}_{2}$ and MoS${}_{2}$-hBN structures. They are much smaller than the one at ℏω= 8 meV and result from shifts of the phonon mode energy caused by the interlayer interactions.

Since coupled electro-thermal simulations were performed, the obtained non-equilibrium phonon population can be converted into an effective lattice temperature with the following equation:

(12) $$\begin{array}{cc} N_{ph}^{tot}\left(s\right)= & \sum_{q_{z}}\sum_{j\in s}\int \frac{d\hbar \omega }{2\pi }LDOS\left({\vec{r}}_{j},\omega ,q_{z}\right)N_{ph}\left(T_{eff}\left(s\right),\omega \right)\\ \; & =i\sum_{q_{z}}\sum_{j\in s}\int \frac{d\hbar \omega }{2\pi }tr\left\{D_{jj}^{<}\left(\omega ,q_{z}\right)\right\}. \end{array}$$

In Equation (12), $N_{ph}^{tot}\left(s\right)$ is the phonon population in the s−th orthorhombic transport unit cell, and the index *j* runs over all atoms in this cell. The phonon population can either be calculated from the lesser phonon Green’s function or by assuming an equilibrium Bose–Einstein distribution function $N_{ph}\left(T_{eff}\left(s\right),\omega \right)$, with an effective lattice temperature $T_{eff}\left(s\right)$ at position *s*. This approach requires the knowledge of the local density-of-states (LDOS) of phonons, which can be derived from:

(13) $$LDOS\left(R_{j},\omega ,q_{z}\right)=i\cdot tr\left\{D_{jj}^{>}\left(\omega ,q_{z}\right)-D_{jj}^{<}\left(\omega ,q_{z}\right)\right\}.$$

**Figure 10:**
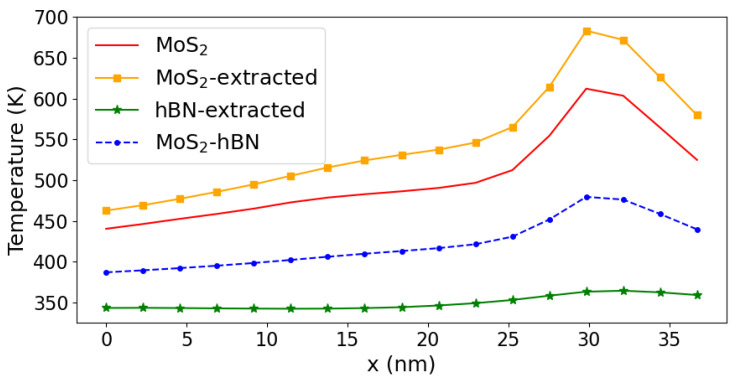
Effective lattice temperature $T_{eff}$ as a function of the position along the transport direction (*x*-axis) in DFT domain 1 (solid red curve) and DFT domain 2 (dashed blue line with dots). The contributions coming from the different layers of the heterostructure are shown as a green line with stars for hBN and as an orange line with squares for MoS${}_{2}$.

Here, $R_{j}$ refers to the position of atom *j*. As all quantities in Equations (10) and (11) have an atomic resolution, an effective temperature can be computed for each layer separately. By doing so, it is possible to determine the impact of the hBN layer on self-heating and on the thermal properties of MoS${}_{2}$ FETs. The effective temperature as computed with Equation (12) is reported in Figure 10. As expected, all curves exhibit a peak located around the position where the phonon current vanishes in Figure 7a,b, i.e., at the interface between the channel and the drain side, where the electric field reaches its maximum. If we consider the whole MoS${}_{2}$ and MoS${}_{2}$-hBN systems, it can be seen that the inclusion of the hBN dielectric layer leads to a lower effective temperature, between 50 and 100 K on average. However, concluding that the presence of an oxide layer is beneficial when it comes to Joule heating would be premature. If one temperature is computed for MoS${}_{2}$ and another one is computed for hBN by separating their respective phonon population, it appears that the semiconductor is hotter than when the hBN layer is not considered in the DFT domain. A temperature increase by 20 to 50 K can be noticed. Its origin can be traced back to the fact that the presence of hBN leads to the creation of addition phonons at ℏω=8meV. The breathing mode raises the MoS${}_{2}$ and hBN temperature by about the same amount, but since this is the only mode that is excited in hBN, the heating effect in that region is less pronounced. Practically, the influence of dielectric layers around 2D semiconductor channels is twofold. On one hand, electrons can emit or absorb additional phonon modes that come from these oxides and that do not exist in pure 2D materials. A higher lattice temperature results from these processes, as demonstrated here. On the other hand, phonons could escape from the transistor active region through the dielectric layer provided that they are attached to a metallic contact or to a heat sink. Such mechanisms could not be taken into account in our simulations. They could counterbalance the temperature increase highlighted in Figure 10.

## 4. Conclusions

We studied the influence of an hBN dielectric on the electrical and thermal behavior of a MoS${}_{2}$ single-gate field-effect transistor. Due to computational reasons, only a single hBN layer could be incorporated into the DFT domain. It was observed that the inclusion (or not) of that layer has almost no impact on the “current vs. voltage” characteristics of the considered device, both in the quasi-ballistic limit of transport (when electron–phonon scattering is treated in a phenomenological way) and when the electron and phonon populations are driven out of equilibrium. However, by examining the phonon modes that interact the most with electrons, it was found that a hybrid mode is induced by the MoS${}_{2}$-hBN coupling at ℏω=8meV, which increases the scattering rate around that energy. Consequently, self-heating becomes more important in the semiconductor channel of the MoS${}_{2}$-hBN system, although the overall temperature is lower than when hBN is absent from the DFT domain. In addition, no phonon with an energy larger than 60 meV interacts with the electron population, i.e., no pure hBN phonon mode is excited, thus confirming the suppression of surface optical scattering in MoS${}_{2}$ transistors encapsulated in hBN that was highlighted in Ref. [15].

**Figure 11:**
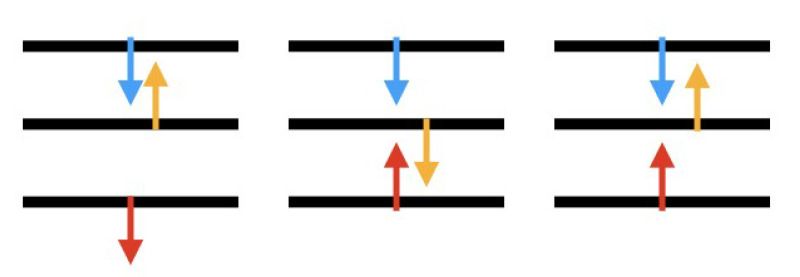
Sketch of the possible breathing modes when more than two atomic planes are considered.

Adding more hBN layers into the DFT domain to model more realistic situations would be the next, most natural step. By doing so, hybrid phonon modes might manifest themselves and affect the electron–phonon scattering rate and the transport properties of MoS${}_{2}$ FETs. In particular, we believe that additional phonon modes that modify the interlayer distance, as the breathing mode, will play an important role by inducing penetration of the MoS${}_{2}$ electronic wave functions into the hBN dielectric. When more than two layers are stacked on top of each other, more out-of-plane vibrations with different phases between the atomic planes are possible, as schematized in Figure 11 for the case of three layers.

The challenge will be to determine the phonon dispersion of the extended MoS${}_{2}$-hBN structure, which might be computationally extremely intensive. As quantum transport simulations are even more demanding, one solution might be to restrict ourselves to the calculation of the phonon spectrum, identify hybrid modes with a breathing character in it, evaluate their scattering strength, and plug the result into a “deformation potential”-like model [67].

## Data Availability

The data presented in this study are openly available in Material Cloud, reference number doi:10.24435/materialscloud:7f-5t.
